# P387: Comparison of antiseptic solutions effectiveness in the hospital environment

**DOI:** 10.1186/2047-2994-2-S1-P387

**Published:** 2013-06-20

**Authors:** A Spyrou, P Vlachos

**Affiliations:** 1 Coronary Care Unit, Onassis Cardiac Surgery Center, Athens, Greece; 2 Cardiac Surgery ICU, Onassis Cardiac Surgery Center, Athens, Greece

## Introduction

The use of antiseptic solutions began in the middle of 19^th^ century in order to protect the spread of cross infections. Nowadays, the use of antiseptic solutions has been proved crucial for skin and hand antisepsis in the hospital environment. There are several antiseptic agents which should be used in every clinical intervention in order to reduce the risk of cross infections.

## Objectives

The comparison between antiseptic solutions effectiveness in the hospital environment.

## Methods

Scientific data bases (PubMed, Cochrane Library) have been used to review articles which compare the effectiveness of various antiseptic agents in the hospital environment. Our search limitations were published research articles, meta-analysis studies and systematic reviews. The articles have been published in English since 2000.

## Results

Every solution containing antiseptic agents has different reaction to the bacterial flora of the skin. Combining different antiseptic agents and solutions seems to have better results in hand and skin antisepsis. Also, alcohol enhances antibacterial effectiveness when present to antiseptic solutions.

## References

In order to prevent nosocomial infections it is important to learn how to choose and implement an antiseptic solution. The development of antiseptic resistance in specific agents is an emerging problem which should be carefully investigated.

## Disclosure of interest

None declared.

